# Next-Generation Water Treatment: Exploring the Potential of Biopolymer-Based Nanocomposites in Adsorption and Membrane Filtration

**DOI:** 10.3390/polym15163421

**Published:** 2023-08-16

**Authors:** Haradhan Kolya, Chun-Won Kang

**Affiliations:** Department of Housing Environmental Design, Research Institute of Human Ecology, College of Human Ecology, Jeonbuk National University, Jeonju 54896, Republic of Korea

**Keywords:** biopolymer-based nanocomposite, water treatment, adsorption, membrane filtration, graphene oxide, carbon nanotubes, nanoclays

## Abstract

This review article focuses on the potential of biopolymer-based nanocomposites incorporating nanoparticles, graphene oxide (GO), carbon nanotubes (CNTs), and nanoclays in adsorption and membrane filtration processes for water treatment. The aim is to explore the effectiveness of these innovative materials in addressing water scarcity and contamination issues. The review highlights the exceptional adsorption capacities and improved membrane performance offered by chitosan, GO, and CNTs, which make them effective in removing heavy metals, organic pollutants, and emerging contaminants from water. It also emphasizes the high surface area and ion exchange capacity of nanoclays, enabling the removal of heavy metals, organic contaminants, and dyes. Integrating magnetic (Fe_2_O_4_) adsorbents and membrane filtration technologies is highlighted to enhance adsorption and separation efficiency. The limitations and challenges associated are also discussed. The review concludes by emphasizing the importance of collaboration with industry stakeholders in advancing biopolymer-based nanocomposites for sustainable and comprehensive water treatment solutions.

## 1. Introduction

Water treatment is a critical global challenge as the demand for clean and safe water rises [1]. With increasing pollution, contamination, and scarcity of freshwater resources, innovative materials and technologies are crucial to addressing these pressing issues [2]. Industrial effluents contain a wide range of pollutants that adversely affect human health when they enter the environment and come into contact with humans through various pathways (in detail in Table 1). Traditionally, water treatment has relied on several methods to ensure the purification of water for various applications, such as coagulation/flocculation [3,4], sedimentation [5,6], filtration [7], disinfection [8], adsorption [9], membrane filtration [10,11]. Although these traditional water treatment methods have specific advantages, they also have certain disadvantages. Coagulation and flocculation [12] effectively reduce turbidity and remove suspended particles, but they may produce a significant amount of toxic sludge, which requires proper disposal. Sedimentation can reduce water treatment costs and the need for additional chemicals but cannot effectively settle smaller particles or remove microbes [13]. Filtration methods, such as sand filtration, may not completely eliminate pathogens and require frequent cleaning and maintenance of filters [14]. Additionally, chlorination is widely used to kill germs, but it is a potent biocide and unsafe [15]. Advanced oxidation techniques offer fast reaction rates and reduced toxicity of organic pollutants, but they are expensive and require careful dose calculations due to their complex chemistry [16]. Ion exchange is an eco-friendly method of neutralizing acidic or basic water, but it faces fouling, bacterial and chlorine contamination, and inadequate regeneration [16]. Anaerobic treatment offers a low-energy and environment-friendly approach but cannot remove inorganic components and generates volatile acids [17]. Microbial treatment is another environment-friendly method used to remove pollutants from water, but it has a short lifespan, is costly, and can result in fouling and low production rates [18].

In contrast, adsorption and membrane filtration stand out for their effectiveness and versatility [19,20]. Adsorption offers high removal efficiency [21], selectivity [22,23], and the potential for regeneration, while membrane filtration provides a reliable method for removing suspended solids, pathogens, and dissolved substances [24]. Therefore, adsorption and membrane filtration technologies have played a crucial role in developing modern water treatment processes, ensuring the delivery of clean and safe drinking water to communities worldwide. Polymers play a crucial role in the enhancing performance and reducing the treatment costs of water treatment methods, particularly in adsorption and membrane filtration processes. Synthetic polymers have traditionally dominated these technologies due to their effectiveness. However, concerns about the biodegradability of synthetic polymers have prompted interest in bio-polymer-based adsorbents and membranes. Bio-polymer-based adsorbents or membranes offer an attractive alternative due to their environmentally friendly nature [25]. Moreover, researchers have recently turned their attention to biopolymeric nanocomposite materials, which are promising for various water treatment applications [26,27]. These materials combine the advantages of biopolymers derived from renewable resources with the unique properties of nanofillers, resulting in enhanced performance and efficiency [28]. Biopolymers, such as chitosan, alginate, cellulose, and starch, offer several advantages in water treatment due to their biocompatibility, biodegradability, and versatility [29,30]. They exhibit excellent adsorption capacities [31], flocculation abilities [32], and antimicrobial properties [33], making them suitable for various water treatment processes [34]. However, incorporating nanofillers into biopolymers has opened up new opportunities for improving their performance and expanding their applications [35]. Nanocomposite materials, composed of biopolymers and nanofillers, possess enhanced mechanical strength, increased surface area, improved stability, and tailored functionalities [36,37]. Nanofillers, such as nanoparticles (e.g., silver, titanium dioxide, zinc oxide, copper oxide, iron oxide, etc.), nanoclays, graphene oxide, and carbon nanotubes, contribute unique properties such as high surface area, catalytic activity, photocatalytic properties, and antimicrobial effects [38,39,40]. These synergistic combinations enable biopolymeric nanocomposites to tackle specific water treatment challenges [41], such as removing heavy metals [42], organic pollutants [43], and microorganisms [44]. The synthesis and characterization of biopolymeric nanocomposites have witnessed significant advancements in recent years [45]. Various methods, including solution casting, in situ polymerization, and electrospinning, have been employed to fabricate nanocomposites with controlled structures and properties [46]. Characterization techniques, such as X-ray diffraction (XRD), scanning electron microscopy (SEM), and thermogravimetric analysis (TGA), provide valuable insights into their morphology, crystallinity, thermal stability, and mechanical properties [47,48].

This review article offers a comprehensive exploration of the emerging field of biopolymeric nanocomposite materials for water treatment applications. It focuses on the synergistic combination of biopolymers and nanofillers, providing insights into their fabrication, characterization, and specific applications in adsorption and membrane filtration. The article also addresses challenges, future perspectives, and the potential to revolutionize water treatment technologies.

**Table 1:** Details about pollutants, source of pollutants, and effects on human health.

Sr. No.	Type Pollutants	Chemical Compositions/ Formula	Sources	Effects	Ref.
1.	Kaolin clay	Al_2_Si_2_O_5_ (OH)_4_	Industrial effluents from Porcelain Rubber Paper Paint Many other products	Water with kaolin clay causes Lung cancer Pneumoconiosis Mesothelioma Aquatic-life problems	[49,50]
2.	Iron ore slimes	Fe_2_O_3_, Fe_3_O_4_, 2Fe_2_O. 3H_2_O, FeCO_3_ and FeS_2_	Washing liquid from Sponge iron industries Steel industries	Water with iron slimes Creates a brown color Damages the reproductive system of aquatic life Degrades the supercoiled DNA	[51,52,53]
3.	Coal	C, H, O, N, S	Coal mining Coal plants Thermal power plants Fly ash Coal bed methane Natural resources	Coal water creates Cancer Lung diseases Heart damage Birth defects	[54,55,56]
4.	Silicon dioxide	SiO_2_	Wastage from industries of Electrical items Solar cell production	Silica in drinking water causes: Alzheimer’s disease Cognitive impairment Dementia	[57,58,59,60]
5.	Copper	Cu	Wastewater from industries of Electrical conductors Heat conductors Building materials Jewelry Steel Alloy metals	Copper intoxication Inhibits the growth of phytoplankton Produces hydroxyl radicals Effects on proteins, lipids, central nervous system, and DNA Cognitive impairment	[61,62,63,64,65]
6.	Nickel	Ni	Effluents from Stainless steel Nickel-processing industries Amalgam-processing industries	Nickel leads to effects such as Disordered lung function Non-remitting bronchitis Neurotoxic Genotoxic Regenerative poisonous Nephrotoxic Embryo toxic Teratogenic	[66,67]
7.	Zinc	Zn	Industrial effluents from Brass Corrosion-resistant zinc plating of iron Amalgam steel Zinc processing units	High concertation of zinc causes Sickness Vomiting Epigastric irritation Laziness Toxicity to plants and invertebrates	[68,69]
8.	Lead	Pb	Wastewater from Lead–acid batteries Atomic power plants Electrodes making	Lead causes mostly Anemia Hypertension Kidney and bone damage Disruption of the nervous system Sperm damage	[70,71]
9.	Mercury	Hg	Wastage from Coal mining Industrial uses Fossil fuel combustion Mining	Mercury has dangerous effects on The central nervous system Cardiovascular system Kidney and bone Skin	[72]
10.	Chromium	Cr	Effluents from Chromite mineral Stainless steel industries Chemical industries Leather industries	Cr (VI) is dangerous and creates Skin irritation Stomatitis Pneumonitis Inhibits the growth of oceanic life	[73,74]
11.	Arsenic	As	Industrial wastewater from Wood preservatives Pesticides Wood processes Agrochemicals Natural resources	Long ingestion of arsenic (III) (>10 ppb) can lead to Arsenicosis Hemolysis Cancer Neurological disorders and painful patches on the hand/feet	[75,76]
12.	Malachite green (MG)	C_23_H_25_Cl_2_	Industrial effluents from Textile dye Wools Plastic materials Calfskin packaging Leather and rubber Paint	High concentration of MG Cuts off sunlight from water Affects the photosynthesis of sea-going plants Disorders the DNA sequence Causes hepatitis Vomiting	[77]
13.	Congo red (CR)	C_32_H_22_N_6_Na_2_O_6_S_2_	Wastewater from Color manufacturer Printing and dyeing machines Paper and plastic productions	High concentration of CR creates Presenile dementia Degenerative disorder diseases Mutagenesis	[78,79]
14.	Methylene blue (MB)	C_16_H_18_ClN_3_S	Effluents from Textile manufacturers Leather and paper manufacturing Color manufacturing industries	MB creates mostly Headache Hypertension Nausea Forgetfulness Dyspepsia Red blood cell breakdown Allergic reactions Central nervous system toxicity	[80,81]
15.	Reactive Black 5 (RB5)	C_26_H_21_N_5_Na_4_O_19_S_6_	Wastewater from Textiles Dye houses Paper printing	Presence of RB5 in water bodies causes Aesthetic problems Obstruction of sun light penetration Oxygen transfer into water Effects on aquatic life	[82,83]
16.	Reactive Blue 4 (RB4)	C_23_H_14_Cl_2_N_6_O_8_S_2_	Effluent from Textile units Plastic manufacturing Printing houses	Presence of RB4 in water bodies causes Pollution in water bodies Effects on aquatic life Mutagenesis	[84,85]
17.	Reactive Blue 29 (RB29)	C_29_H_15_Cl_2_N_5_Na_2_O_9_S_2_	Discharged wastewater from Textile industries Plastic industries	RB29 affects Esthetic merit Light penetration Carcinogenesis	[86]
18.	Reactive Yellow 145 (RY145)	C_28_H_20_ClN_9_Na_4_O_16_S_5_	Wastewater from Paper printing Textile industries Plastic industries	RY145 toxic towards Humans Aquatic life	[87,88]
19.	Pharmaceuticals n: (Ibuprofen: Metformin: Simvastatin: Omeprazole: Fluoxetine: Ciprofloxacin: Amlodipine: Sertraline: Atorvastatin:)	C_8_H_9_NO_2_ C_13_H_18_O_2_ C_4_H_11_N_5_ C_25_H_38_O_5_ C_17_H_19_N_3_O_3_S C_17_H_18_F_3_NO C_17_H_18_FN_3_O_3_ C_20_H_25_ClN_2_O_5_ C_17_H_17_Cl2N C_33_H_35_FN_2_O_5_	Human excretion Improper disposal Pharmaceutical industries	Antibiotic resistance Endocrine disruption Allergic reactions Drinking water contamination	[89,90]
20.	Personal Care (Products: Parabens: Triclosan: Benzophenone-3: Phthalates: Octinoxate: Formaldehyde: Sodium Lauryl Sulfate:)	C_9_H_10_O_3_ C_12_H_7_C_l3_O_2_ C_14_H_12_O_3_ C_8_H_4_O_4_ C_18_H_26_O_3_ CH_2_O C_12_H_25_NaO_4_S	Direct disposal into sinks, showers, and toilets Runoff from treated areas (e.g., sunscreen washed off during swimming) Improper disposal and inadequate wastewater treatment	Toxic to aquatic life Endocrine disruption Interfere with hormone function Respiratory irritation	[91]
21.	Microplastics (Polyethylene: Polypropylene: Polystyrene:)	(C_2_H_4_)n (C_3_H_6_)n (C_8_H_8_)n	Wastewater discharge from industries and households. Urban runoff carrying plastic litter. Degradation of larger plastic items in the environment. Release of microfibers from washing synthetic clothing.	Ingestion Neurotoxicity Damage to the digestive tracts of marine animals	[92,93]

## 2. Effects of Pollutants on Human Health

The effects of pollutants on human health are of significant concern in environmental health. Table 1 provides an overview of the different sources of pollutants found in industrial effluents and highlights their specific effects on human health. These pollutants include heavy metals, organic compounds, toxins, and other harmful substances. Each pollutant reveals unique health effects depending on its chemical properties and the extent and duration of exposure. The health effects of pollutants can vary extensively, ranging from acute to chronic and long-term health implications. Acute effects may include immediate symptoms such as nausea, vomiting, respiratory distress, or skin irritation. Chronic exposure to pollutants over a prolonged period can lead to various health problems, including respiratory disorders, neurological disorders, cardiovascular diseases, reproductive disorders, and certain cancers. Understanding and monitoring the sources and levels of pollutants in industrial effluents and using improved technologies to minimize human exposure and mitigate potential health risks is crucial.

## 3. Biopolymers

Biopolymers have expanded significant attention in water treatment due to their unique properties and multipurpose applications [94]. They are derived from renewable sources, making them environmentally friendly alternatives to synthetic polymers. Biopolymers are generally non-toxic, biocompatible, and biodegradable, minimizing the potential for secondary pollution. Moreover, their flexibility allows for modifications and functionalization to improve performance and target specific contaminants [95]. This section will explore the different types of biopolymers commonly used in water treatment and their effectiveness in various processes.

**Figure 1:**
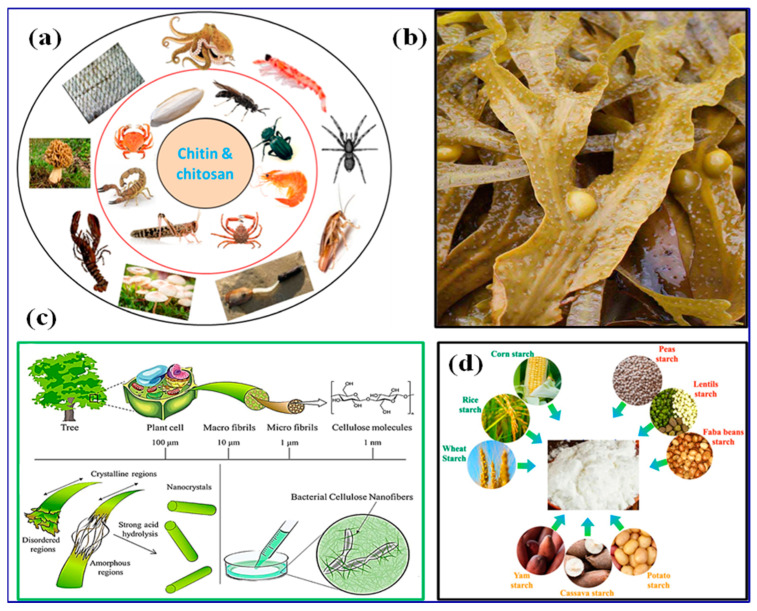
Schematic representation of the source of (**a**) chitosan [97]. Copyright 2022; reproduced with permission from Springer Nature, (**b**) alginate [99]. Copyright 2020; reproduced with permission from Intechopen Limited, (**c**) cellulose [103]. Copyright 2021; reproduced with permission from Elsevier B.V. All rights reserved, and (**d**) starch [105]. Copyright 2022; reproduced with permission from the authors, MDPI, Basel.

Chitosan: Chitosan [96], derived from chitin (as shown in Figure 1a) [97], is a broadly studied biopolymer with excellent adsorption capabilities. Its high surface area and positive charge effectively remove heavy metals, dyes, and organic pollutants from water through electrostatic attraction and coagulation. Chitosan-based materials can be used as beads, membranes, and hydrogels for applications like adsorption, filtration, and wastewater treatment.

Alginate: Alginate [98], extracted from seaweed (Figure 1b) [99], is a biopolymer known for its gel-forming properties and biocompatibility. It is commonly used in water treatment for applications such as encapsulation, immobilization of enzymes or microorganisms, and removal of metal ions [100,101,102]. Alginate beads and membranes have shown promise in removing metals and dyes through ion exchange and adsorption mechanisms.

Cellulose: Cellulose, the most plentiful biopolymer on Earth (Figure 1c), has garnered attention for its exceptional mechanical strength, hydrophilicity, and biodegradability. Cellulosic materials have been used for water treatment processes such as adsorption, membrane filtration, and separation techniques. Modified cellulose-based materials, including cellulose nanocrystals and cellulose derivatives, offer higher surface area and adsorption properties, effectively removing contaminants [103,104].

Starch: Starch, derived from crops (Figure 1d) [105], possesses unique properties such as biodegradability, abundance, and low cost. Starch-based materials have been explored for applications such as adsorption, flocculation, and membrane filtration. Modified starches and starch-based hydrogels can potentially remove organic pollutants, dyes, and microorganisms from water [106,107,108].

**Figure 2:**
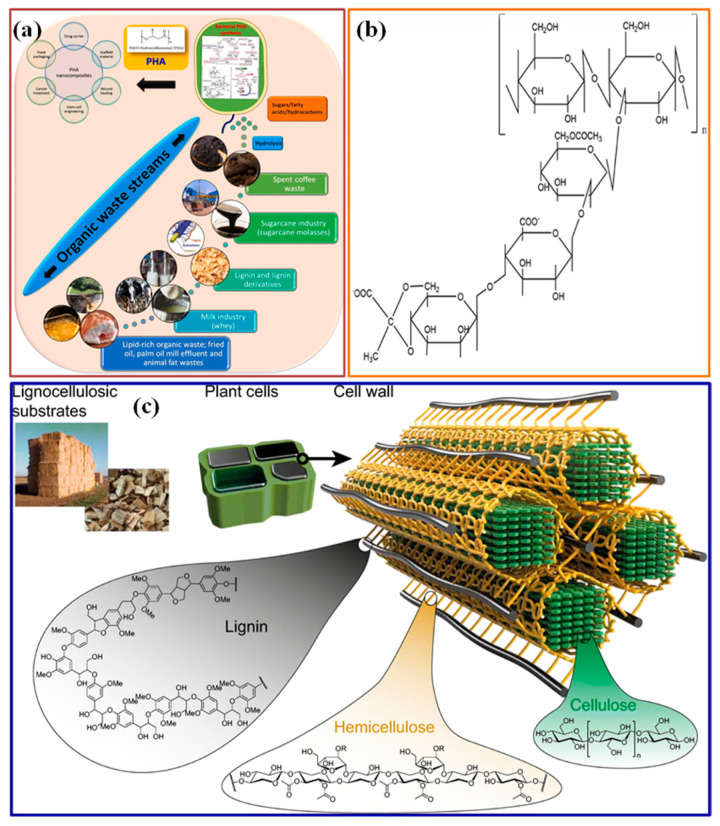
Schematically presentation of source and formula structures of (**a**) PHA [109]. Copyright 2022; reproduced with permission from Elsevier B.V. All rights reserved, (**b**) Xanthan gum [113]. Copyright 2017; reproduced with permission from Wiley-vch Verlag GmbH & Co. KGaA, Weinheim, and (**c**) lignin [116]. Copyright 2022; reproduced with permission from the Author(s).

Poly(hydroxyalkanoates) (PHA): Poly(hydroxyalkanoates) are biodegradable and biocompatible polyesters synthesized by various microorganisms (Figure 2a) [109,110]. PHA-based materials have shown promise in water treatment applications, particularly in removing organic pollutants and heavy metals. PHA films, beads, and membranes have demonstrated efficient adsorption capacities and can be used in adsorption, filtration, and wastewater treatment [111].

Xanthan Gum: Xanthan gum is a polysaccharide produced by the bacterium Xanthomonas campestris [112], as shown in Figure 2d [113]. Its high viscosity and stabilizing properties make it suitable for various water treatment applications [114]. Xanthan gum has been utilized for the flocculation, sedimentation, and stabilization of particles in water and wastewater treatment processes [115].

Lignin: Lignin is a complex and abundant biopolymer found in plant cell walls, as shown in Figure 2e [116]. It is an attractive material for water treatment due to its aromatic structure and adsorption properties [117]. Lignin-based materials, such as lignin derivatives, nanoparticles, and membranes, can potentially remove organic pollutants, dyes, and heavy metals from water [118].

However, it is crucial to note that the effectiveness of biopolymers in water treatment can be influenced by factors such as pH, temperature, concentration, and the nature of contaminants. Optimization of operating conditions and the selection of suitable biopolymers are crucial for achieving desired treatment outcomes.

## 4. Biopolymer-Based Nanocomposite

Nanocomposite materials, which combine biopolymers with nanofillers, have emerged as promising candidates for water treatment applications [119]. Parallel to their components, these materials offer enhanced performance, enhanced properties, and increased functionality [120]. In this section, we will explore the concept of nanocomposites and the various types of nanofillers combined with biopolymers for water treatment. Nanofillers can be nanoparticles, nanoclays, nanotubes, or other nanoscale materials, and they contribute specific functionalities such as increased surface area, enhanced mechanical strength, catalytic activity, or antimicrobial properties.

### 4.1. Nano-Fillers for Water Treatment

A wide range of nanofillers can be incorporated into biopolymer matrices for water treatment applications [121]. Here are some commonly used nanofillers:

Nanoparticles: Nanoparticles play a crucial role in water treatment applications, offering various properties and functionalities. Silver nanoparticles (AgNPs) and titanium dioxide nanoparticles (TiO_2_NPs) are well known for their antimicrobial and photocatalytic properties. AgNPs exhibit strong antibacterial and antiviral effects, while TiO_2_NPs can induce photocatalytic reactions to degrade organic pollutants in water [122,123,124]. In addition to these, other nanoparticles—such as zinc oxide nanoparticles (ZnONPs) with their photocatalytic and antimicrobial properties [125]; iron oxide nanoparticles (FeOxNPs) for adsorption, catalytic degradation of dyes, and magnetic separation [126,127]; copper nanoparticles (CuNPs) for antimicrobial applications [128]; carbon-based nanoparticles like carbon nanotubes (CNTs) and graphene oxide (GO) for adsorption of contaminants [129]; cerium oxide nanoparticles (CeO_2_NPs) for catalytic degradation [130]; and hybrid nanoparticles with tailored functionalities—are also being extensively studied [131]. Each type of nanoparticle brings unique advantages to water treatment processes, and ongoing research aims to harness their potential for efficient and sustainable water purification. 

Nanoclays: Nanoclays, such as montmorillonite and halloysite nanotubes, have great potential as biopolymer composites for water purification applications [132,133]. These nanoparticles have distinct features that improve the composites’ ability to remove pollutants from water. Incorporating montmorillonite nanoclay into biopolymer matrices such as chitosan or alginate, for example, has been shown to increase the adsorption capacity and selectivity of the composites towards heavy metals, organic contaminants, and colors [134]. Nanoclays’ high surface area and ion exchange capacities allow for effective pollutant adsorption and removal via surface complexation, ion exchange, and intercalation. Furthermore, the layered structure of nanoclays creates a difficult path for pollutants to diffuse, increasing purification efficiency.

**Figure 3:**
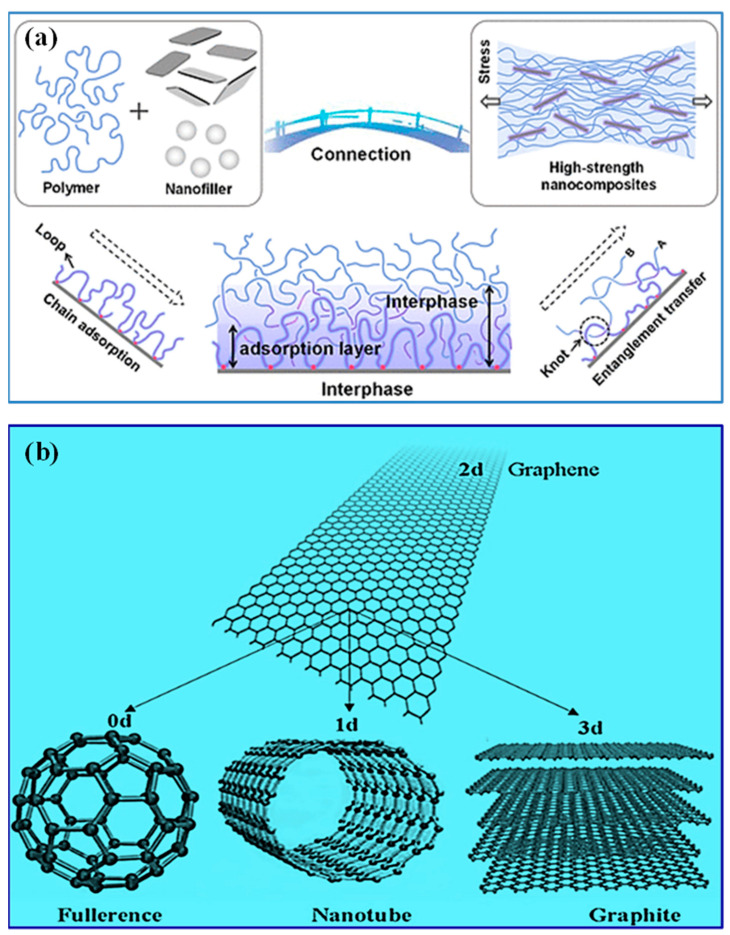
Schematic representation of (**a**) incorporation of nanoclays into the polymer matrix [136]. Copyright 2022; reproduced with permission from the authors, and (**b**) structures of carbonaceous nanofillers [145]. Copyright 2021; reproduced with permission from MDPI, Basel.

On the other hand, halloysite nanotubes have a hollow tubular shape that can be used for the controlled release of antimicrobial agents or active species, providing additional capabilities for water treatment. Combining nanoclays with biopolymers improves adsorption and removal capacities and increases the composite materials’ mechanical strength and stability, making them appropriate for filtering membranes and adsorbent media [135]. Developing nanoclay-based biopolymer composites for water purification has the potential to alleviate water pollution issues while also encouraging sustainable and effective treatment methods. Figure 3a depicts a schematic of integrating nano-filler into the polymer matrix [136].

Carbon Nanotubes: Carbon nanotubes (CNTs) possess unique properties, including exceptional mechanical strength, high electrical conductivity, and excellent thermal stability, making them highly attractive for a variety of applications in water treatment. In the context of nanocomposites for water purification, CNTs have revealed promising results, particularly in membrane technology [137]. When incorporated into polymeric matrices, CNT-based nanocomposites have shown improved performance in water treatment compared to traditional nanomaterials and clay composites. For example, CNT-reinforced polymeric membranes exhibit better permeability, allowing for increased water flux rates while maintaining the practical separation of contaminants. This improved permeability is attributed to the unique structure of CNTs, which provide a network of interconnected nanochannels within the membrane, facilitating the flow of water molecules [138,139].

Moreover, CNTs can enhance the selectivity of membranes by acting as molecular sieves, effectively blocking the passage of specific contaminants based on size or charge. This selectivity is particularly advantageous in removing small organic molecules or heavy metal ions from water sources. Additionally, the high aspect ratio and surface area of CNTs contribute to their adsorption capacity, allowing for the effective removal of organic pollutants and dyes from water [140,141].

It is worth noting that while nanoclays offer their own set of advantages in water treatment, such as ion exchange capacity and high surface area, CNT-based nanocomposites provide distinct benefits. The exceptional mechanical and electrical properties of CNTs contribute to the overall performance and durability of the composite membranes. Moreover, the unique nanochannel structure and fouling resistance properties of CNTs set them apart as promising nanomaterials for membrane technology in water treatment.

Graphene and Graphene Oxide: Graphene and graphene oxide (GO) are highly promising carbon-based materials with excellent properties that make them attractive for various applications in water treatment [40]. These two-dimensional carbon structures possess a large surface area, high mechanical strength, and excellent adsorption capabilities contributing to their effectiveness in water purification processes [142]. In nanocomposites for water treatment, graphene- and GO-based materials have shown significant prospective applications, particularly in removing heavy metal ions, organic pollutants, and emerging contaminants from water sources [143]. Their large surface areas provide ample active sites for adsorption, allowing them to effectively capture and remove contaminants through physical interactions [143]. This adsorption capacity is especially beneficial for removing heavy metal ions, organic compounds, and micropollutants. Graphene-based nanocomposites have established superior adsorption performance compared to traditional nanomaterials, clay composites, and carbon nanotubes (CNTs) [144]. The unique structure of graphene and GO, consisting of a single layer of carbon atoms arranged in a hexagonal lattice, offering a highly accessible and reactive surface for interaction with contaminants. This results in enhanced adsorption efficiency and removal rates.

Moreover, graphene-based nanocomposites reveal exceptional mechanical strength, improving the membranes’ durability and lifespan. Furthermore, the chemical adaptability of graphene and GO allows for functionalization and modification, tailoring their properties for specific water treatment applications. Surface modifications can develop selectivity, improve stability, or introduce additional functionalities to the nanocomposites. This flexibility in functionalization expands the potential applications of graphene-based nanocomposites in water treatment. While nanoclays, CNTs, and other nanomaterials offer their unique advantages in water treatment, graphene- and GO-based nanocomposites stand out due to their exceptional properties, including large surface area, high mechanical strength, excellent adsorption capabilities, and electrical conductivity [144]. These properties collectively contribute to their superior performance in water purification processes. The structures of carbonaceous nanofillers are shown in Figure 3b [145].

### 4.2. Synthesis and Characterization

The synthesis of biopolymeric nanocomposites embroils the incorporation of nanofillers, such as nanoparticles or nanofibers, into a biopolymer matrix. Various techniques can be employed to disperse the nanofillers within the biopolymer matrix, depending on the preferred structure and properties of the nanocomposite.

**Figure 4:**
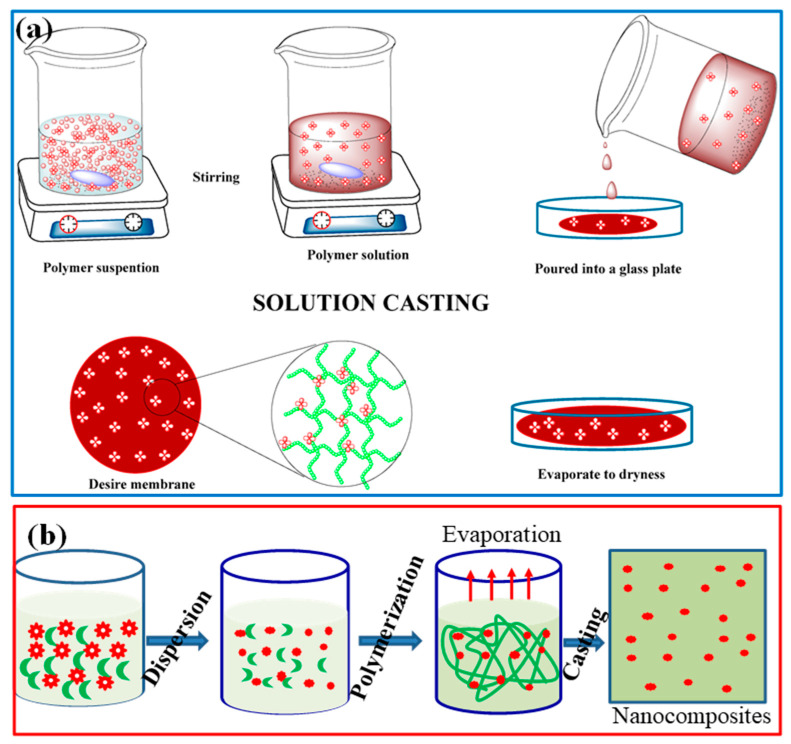
Schematic presentation of polymer nanocomposites synthesis process (**a**) solution casting [146]. Copyright 2017; reproduced with permission from the authors, MDPI, Basel. and (**b**) In situ polymerization method.

Solution casting: One commonly used method is solution casting, in which the biopolymer is dissolved in a suitable solvent and the nanofillers are dispersed in the polymer solution. The mixture is then cast into a mold or onto a substrate and allowed to dry, resulting in a solid nanocomposite material (Figure 4a) [146]. Solution casting allows for specific control over the nanofillers’ dispersion and the nanocomposite’s overall composition.

In situ polymerization: In situ polymerization is another approach to synthesizing biopolymeric nanocomposites. In this method, the biopolymer is synthesized or partially polymerized in the presence of the nanofillers (Figure 4b). The nanofillers act as nucleation sites for the polymerization process, forming a nanocomposite structure [147]. This method offers good interfacial bonding between the nanofillers and the polymer matrix, enhancing mechanical properties and stability.

**Figure 5:**
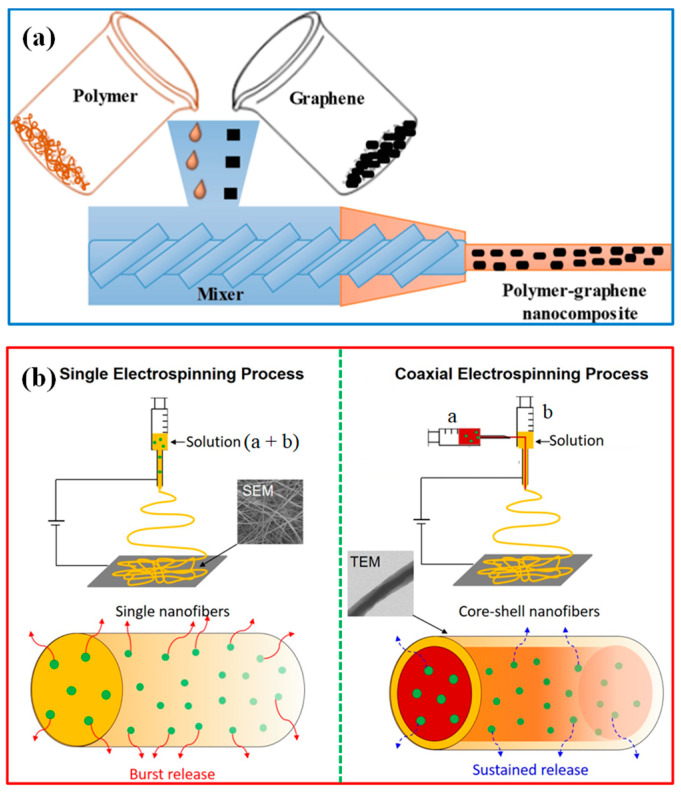
Schematic of (**a**) melt mixing method [148]. Copyright 2020; reproduced with permission from Springer Nature Switzerland AG. and (**b**) single electrospinning and coaxial electrospinning process [149]. Copyright 2021; reproduced with permission from the authors.

Melt blending: Melt blending is a technique commonly employed for thermoplastic biopolymers. It involves melting the biopolymer and adding nanofillers, followed by mixing and solidification to obtain a homogeneous nanocomposite material (Figure 5a) [148]. Melt blending is relatively simple and scalable, making it suitable for the large-scale production of biopolymeric nanocomposites.

Electrospinning: Electrospinning is a particular technique used to fabricate nanofiber-based nanocomposites. In this method, a polymer solution containing the nanofillers is electrostatically spun into ultrafine fibers. The resulting nanofibers form a greatly porous structure with a higher surface area, making them suitable for applications such as filtration membranes. Electrospinning allows for precise control over the nanofiber diameter and distribution, resulting in tailored properties for water treatment applications. A schematic of the single electrospinning process and (b) the coaxial electrospinning process is shown in Figure 5b.

**Figure 6:**
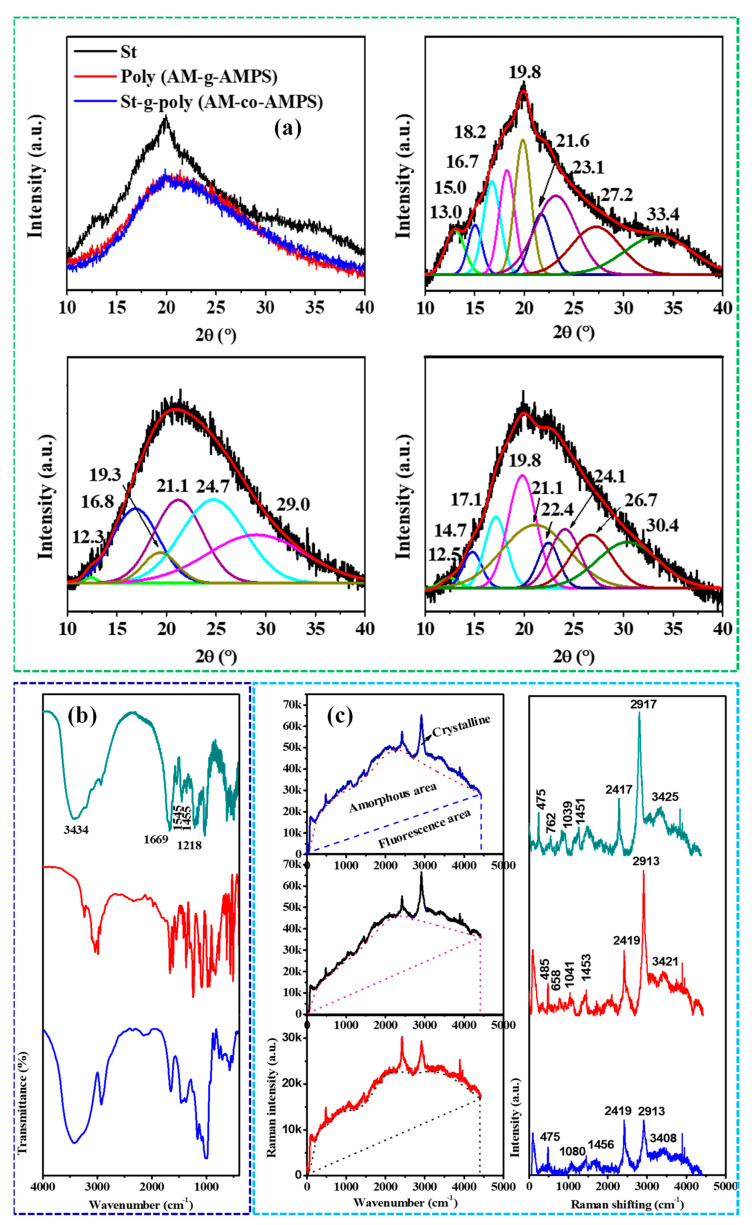
Spectra with their background corrections and deconvolution for crystallinity study (**a**) XRD, (**b**) ATR-FTIR, and (**c**) Raman [94]. Copyright 2022; reproduced with permission from Elsevier B.V. All rights reserved.

Characterization techniques play a crucial role in understanding polymer nanocomposites’ structure, morphology, thermal stability, and mechanical properties [150]. Among the commonly used techniques, X-ray diffraction (XRD) provides valuable information about polymer or polymer nanocomposite materials’ crystalline structure, phase composition, and degree of crystallinity. Furthermore, integrating spectroscopic techniques such as Fourier-transform infrared spectroscopy (FTIR) and Raman spectroscopy provides valuable chemical information about the polymers’ and polymer nanocomposites’ functional groups and molecular interactions. Generally, the Raman intensity varies directly with the size of the crystalline region. The Raman spectrum was also used to evaluate the change in crystal structures of polymers [94]. Examples of FTIR, Raman, and XRD spectra are shown in Figure 6.

**Figure 7:**
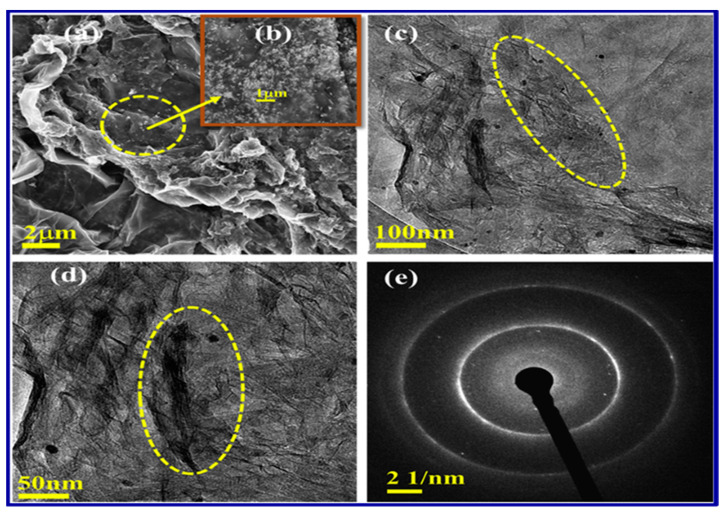
Micrographs of AgNPs/rGO nanocomposite, (**a**,**b**) FESEM, and (**c**,**d**) TEM, and (**e**) SAED pattern image [129]. Copyright 2019; reproduced with permission from Elsevier Ltd. All rights reserved.

Scanning electron microscopy (SEM) and transmission electron microscopy (TEM) are powerful imaging techniques that allow for the visualization of the nanocomposite’s surface and internal structure at different length scales [129]. SEM provides high-resolution images of the material’s surface, enabling the examination of particle distribution, agglomeration, and interfacial interactions (as shown in Figure 7). Conversely, TEM provides an even higher resolution, allowing for a detailed examination of individual nanoparticles and their dispersion within the polymer matrix.

**Figure 8:**
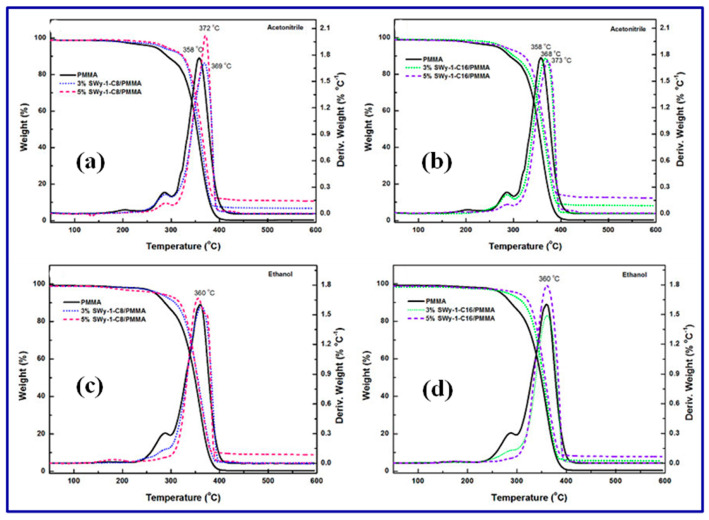
(**a**) TGA curves for PMMA, (**b**) DTGA curves for nanocomposites in acetonitrile medium and (**c**,**d**) in ethanol medium [151]. Copyright 2013; reproduced with permission from the authors.

Thermal analysis techniques, such as thermogravimetric analysis (TGA) and differential scanning calorimetry (DSC), provide insights into polymer nanocomposites’ thermal stability and behavior. TGA measures the weight loss of the sample as a function of temperature, providing information about its thermal degradation and decomposition. DSC measures the heat flow associated with phase transitions, melting points, and glass transition temperatures, giving valuable data on the thermal properties and crystallinity of the nanocomposite (as shown in Figure 8).

Advancements in characterization techniques have led to improved resolution, sensitivity, and quantitative analysis capabilities. For example, advanced microscopy techniques such as atomic force microscopy (AFM) and high-resolution TEM (HRTEM) have allowed for more precise characterization of nanoscale structures and interfaces. X-ray photoelectron spectroscopy (XPS), also known as electron spectroscopy for chemical analysis (ESCA), is another crucial characterization technique for analyzing polymer nanocomposites.

**Figure 9:**
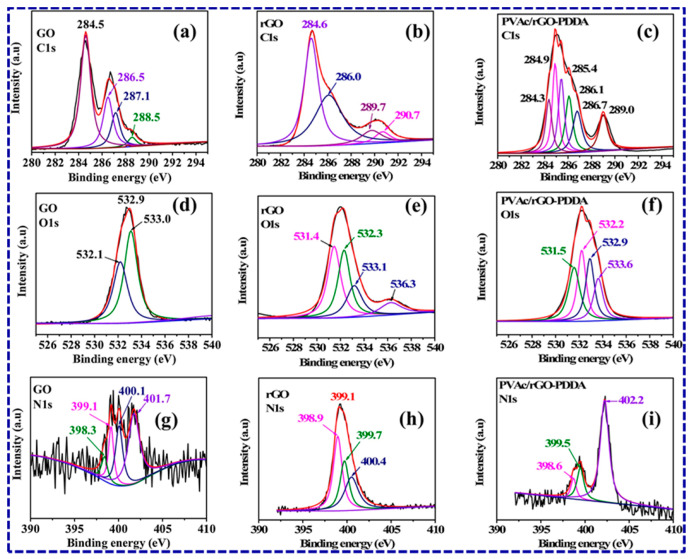
The XPS C1s spectra of GO, rGO, and rGO/PVAc-PDDA are shown in (**a**–**c**). The XPS O1s spectra of GO, rGO, and rGO/PVAc-PDDA are shown in (**d**–**f**), while the XPS N1s spectra of GO, rGO, and rGO/PVAc-PDDA are presented in (**g**–**i**) [152]. Copyright 2021; reproduced with permission from Elsevier B.V. All rights reserved.

XPS analysis is particularly useful for studying the interaction between nanoparticles and the polymer matrix in nanocomposites. It can provide insights into the chemical bonding, charge transfer, and electronic structure at the nanoparticle–polymer interface. This information is crucial for understanding the mechanisms of reinforcement, adhesion, and dispersion of nanoparticles within the polymer matrix (Figure 9).

Combining XPS analysis with other characterization techniques, such as SEM or TEM, allows for a comprehensive understanding of the nanocomposite’s structure, morphology, and surface properties. The information obtained from XPS analysis helps researchers optimize the synthesis parameters, modify the surface chemistry, and tailor the properties of polymer nanocomposites for specific applications.

However, it is essential to exercise caution during polymer nanocomposites’ synthesis and sample preparation to ensure accurate characterization. Factors such as the choice of solvents, processing conditions, and sample handling can influence the dispersion and aggregation of nanoparticles, affecting the final properties of the nanocomposite. Careful control of these parameters is necessary to obtain reliable and reproducible results during characterization.

### 4.3. Applications in Water Treatment

#### 4.3.1. Adsorption

The adsorption process is widely used in water treatment to remove contaminants from water [153]. It involves the attachment of pollutants to the surface of adsorbent material, leading to their separation from the water. The adsorbent material can be granules, pellets, or powder, providing a large surface area for effective adsorption. Several commercial adsorbents are commonly used in water treatment applications [154]. Activated carbon is one of the most widely used adsorbents due to its high adsorption capacity and versatility in removing many contaminants, including organic compounds, odors, and taste-causing substances [155]. Other commercial adsorbents include zeolites, silica gel, and clay minerals, which have specific adsorption properties for different pollutants [154]. Despite their effectiveness, commercial adsorbents have some drawbacks. One of the main limitations is their high cost, specifically in large-scale applications. The regeneration of adsorbents can also be challenging and costly in some cases.

Bionanocomposites as adsorbents have developed as a promising approach in recent years [119]. Bionanocomposites are hybrid materials that combine biopolymers with nanoparticles, offering enhanced adsorption properties [121]. These composites leverage the high surface area and functional properties of nanoparticles along with the biocompatibility and renewable nature of biopolymers. For example, chitosan–clay nanocomposites have garnered significant attention due to their abundant availability, ease of production, and effectiveness as adsorbents. This specific class of composites has demonstrated the ability to quantitatively remove 99% of dyes, metals, and hazardous negatively charged ions from water [156]. In addition, graphene oxide–potato starch composites have been investigated for their ability to remove MB dye from industrial effluents using adsorption. The composite exhibited a significant adsorption potential of approximately 90% [157]. Incorporating GO nanosheets with polysaccharide long chains in the composite created a plywood-like structure with nanocages, enhancing the adsorption of organic dyes [158]. Two types of interactions contribute to the adsorption mechanism: (a) electrostatic interactions between GO and cationic dyes, and (b) π–π stacking interactions between the aromatic moiety of the dye and the delocalized π-electron system of GO.

**Figure 10:**
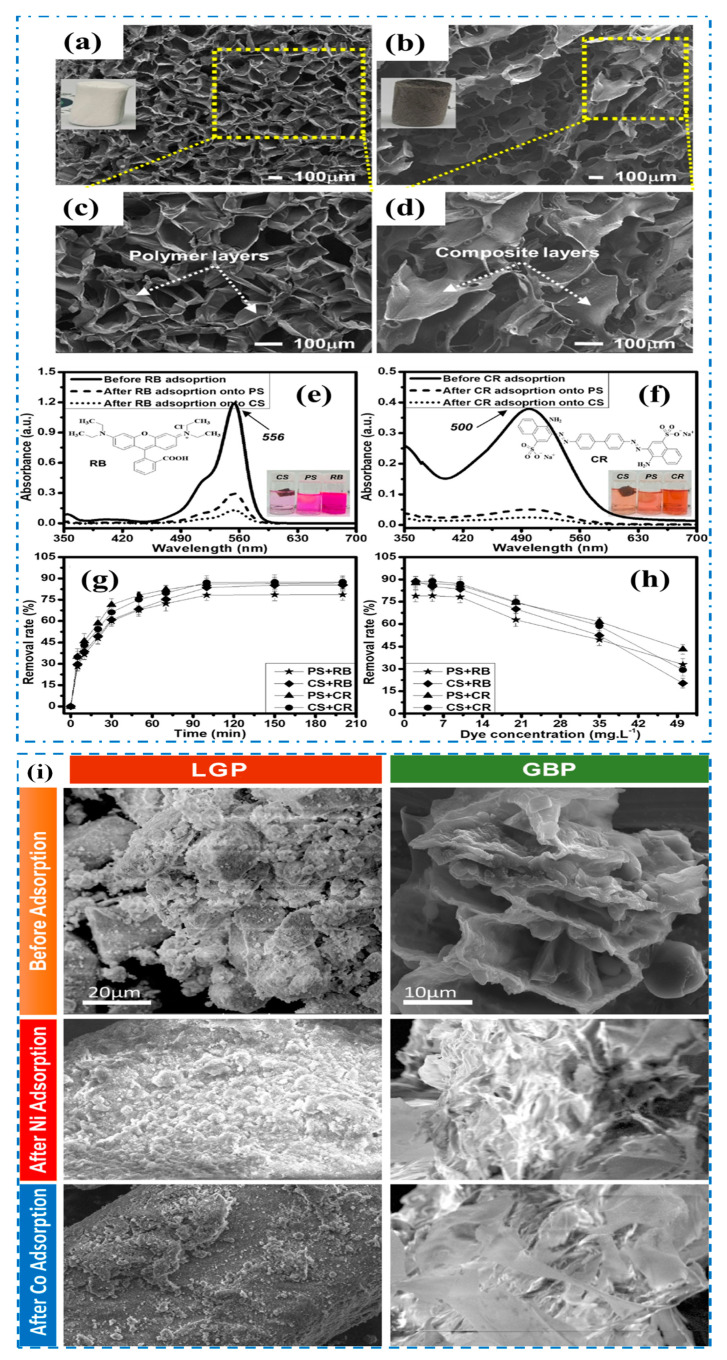
SEM images of biopolymer sponge (**a**,**c**) and composite sponge (**b**,**d**). UV–Vis spectra of different dye solutions before and after adsorption: RB (**e**) and CR (**f**). Dye-adsorbed sponge adsorption studies: adsorptive times (**g**) and dye concentrations (**h**) [162]. Copyright 2022; reproduced with permission from the Chinese Materials Research Society. Published by Elsevier B.V. (**i**) SEM images of laterite-clay-based geopolymer and Grewia biopolymer samples [163]. Copyright 2022; reproduced with permission from the Taiwan Institute of Chemical Engineers. Published by Elsevier B.V. All rights reserved.

Graphene oxide also interacts with heavy metal ions through surface complexation for metal ion adsorption. Graphene oxide–chitosan composites have shown excellent stability and mechanical qualities for wastewater adsorption treatment. Chitosan, a cationic biopolymer with combative amino (―NH_2_) and hydroxyl (―OH) groups, exhibits efficient coagulative abilities for extracting pollutants from aqueous solutions. However, challenges such as higher synthesis costs, pH maintenance, and inefficacy at low concentrations limit the practical use of these materials. Most current research focuses on the adsorption properties of single heavy metal ions, and limited research is available on treating mixtures of heavy metal ions in wastewater [159,160]. AP-g-3D GO composites have also demonstrated efficient adsorption of various organic and inorganic pollutants from water [161]. Chitosan–poly(vinyl alcohol)–graphene oxide cross-linked sponge showed high adsorption capacities for rhodamine B (RB) and Congo red (CR) dyes [162]. The 3D porous structures of the biopolymer sponge and composite sponge enhanced the π–π interaction with the aromatic rings of the dye molecules. The surface morphology of the biopolymer sponge and 3D composite sponge is shown in Figure 10a–d, and the results of dye removal capacity are shown in Figure 10e–h.

A recent study has revealed that combining a geopolymer with a biopolymer enhances the adsorption efficiency of Ni(II) and Co(II) ions in wastewater. The synergistic effect of lateritic geopolymer and Grewia biopolymer, derived from the Tiliaceae family, resulted in the removal of approximately 80% of Ni(II) and Co(II) ions from the wastewater samples. The laterite-clay-based geopolymer is rich in aluminum, iron, and silica oxides, while the Grewia biopolymer consists of simple carbohydrates such as glucose, mannose, arabinose, xylose, and glucuronic acid. The laterite-clay-based geopolymer exhibits a kaolinite platelet structure, whereas the Grewia biopolymer possesses irregularly shaped open pores (Figure 10i), likely contributing to its superior adsorption capabilities [163].

Graphene oxide/chitosan composites have been extensively studied for wastewater adsorption treatment (Table 2). Chitosan, a cationic biopolymer with hydrophobic and biodegradable properties, possesses efficient coagulative ability in extracting pollutants from aqueous solutions. However, the practical use of these materials is limited due to higher synthesis costs, pH maintenance requirements, and inefficiency at low concentrations. The reported research studies provide valuable insights into the adsorption capacities of different graphene oxide/chitosan composites for various pollutants.

For instance, the GO-cl-potato starch bio-composite exhibited a remarkable adsorption capacity of 500 mg/g (90%) for MB dye [157]. Another composite, St-g-poly(AM-co-GO)/hydroxyapatite, demonstrated 297 mg/g adsorption capacity for MG dye [164]. Furthermore, AP-g-3D GO composites showed adsorption capacities for various pollutants, such as tert-butyl hydroquinone, o-nitrophenol, p-aminophenol, and others [161]. The 3D GO-sodium alginate composite achieved an adsorption capacity of 833.3 mg/g for MB dye [165]. In contrast, GO-calcium/alginate composite exhibited adsorption capacities of 163.93 mg/g and 140.85 mg/g for MB dye at different temperatures [166]. Other composites, such as dextrin-g-poly(m-phenylenediamine)-GO, St-g-3DGO, alginate-chitosan hybrid adsorbent, PAA-chitosan and biochar composite, chitosan-GO-Hap composite, and chitosan–GO composites, demonstrating their adsorption capacities for various pollutants [167,168,169,170,171,172]. Additionally, the sodium alginate-CMC-GO-Gd_3_O_3_ composite showed adsorption capacities for Pb, Cr (III), and As (V) [173]. Notably, the chitosan-activated carbon composite achieved a high removal efficiency of 98.7% for Cr (VI) ions [174].

**Table 2:** Summary of bio-based composite polymers as adsorbents for wastewater treatment.

Sl. No	Adsorbent/Flocculent	Pollutants	Results (% or Q_max_ mg/g)	Ref.
1.	GO-cl-potato starch bio-composite	MB	500 mg/g (90%)	[157]
2.	St-g-poly(AM-co-GO)/hydroxyapatite composite hydrogel	MG	297 mg/g	[164]
3.	AP-g-3D GO composites	tert-butyl hydroquinone o-nitrophenol p-aminophenol Hydroquinone p-nitrophenol Neutral red Alizarin red S Pb (II) Mn (II) Cr_2_O_7_^−2^ Cd (II) Cu (II) Nd (III) La (III) Y (III) Yb (III) Yr (III)	22.17 mg/g 36.96 mg/g 116.4 mg/g 16.10 mg/g 36.96 mg/g 44.78 mg/g 39.92 mg/g 84.76 mg/g 7.92 mg/g 13.6 mg/g 17.64 mg/g 30.56 mg/g 25.25 mg/g 12.48 mg/g 16.96 mg/g 23.32 mg/g 30.32 mg/g	[161]
4.	3D GO-sodium alginate	MB	833.3 mg/g, at 303K	[165]
5.	GO-calcium/alginate	MB	163.93 mg/g, at 298 K 140.85 mg/g, at 328 K	[166]
6.	Dextrin-g-poly(m-phenylenediamine)-GO	Pb (II) MB	80 mg/g 76.33 mg/g	[167]
7.	St-g-3DGO	Pb (II) Cu (II) Cd (II) Yb (III) Nd (III)	108.68 mg/g 32.12 mg/g 46.28 mg/g 41.76 mg/g 38.168 mg/g	[168]
8.	Alginate–chitosan hybrid adsorbent	Pb (II)	96.8%, pH 5.0	[169]
9.	PAA–chitosan and biochar-composite	Cu (II), Zn (II), Ni (II), Pb (II), Cd (II), Mn (II), Co (II), and Cr (VI)	80%, pH 2–7	[170]
10.	Chitosan–GO–Hap composite	CR, acid red 1, and reactive red 2	43.06, 41.32, and 40.03 mg/g, pH 2	[171]
11.	Chitosan–GO composites	Reactive black 5 dye	277 mg/g at 25 °C	[172]
12.	GO–Chitosan composite	Cu (II), Pb (II), Cd (II)	60.7, 48.7, 32.3 mg/g, pH 1	[175]
13.	Sodium alginate-CMC-GO-Gd_3_O_3_	Pb (II), Cr (III) and As (V)	29.16, 158.73, and 36.77 mg/g	[173]
14.	Chitosan-activated carbon composite	Cr (VI)	98.7%	[174]
15.	Chitosan–graphene-oxide-dip-coated electrospun nanofiber membrane	MB CR	201 mg/g 152 mg/g	[176]

#### 4.3.2. Magnetic Adsorbents

**Figure 11:**
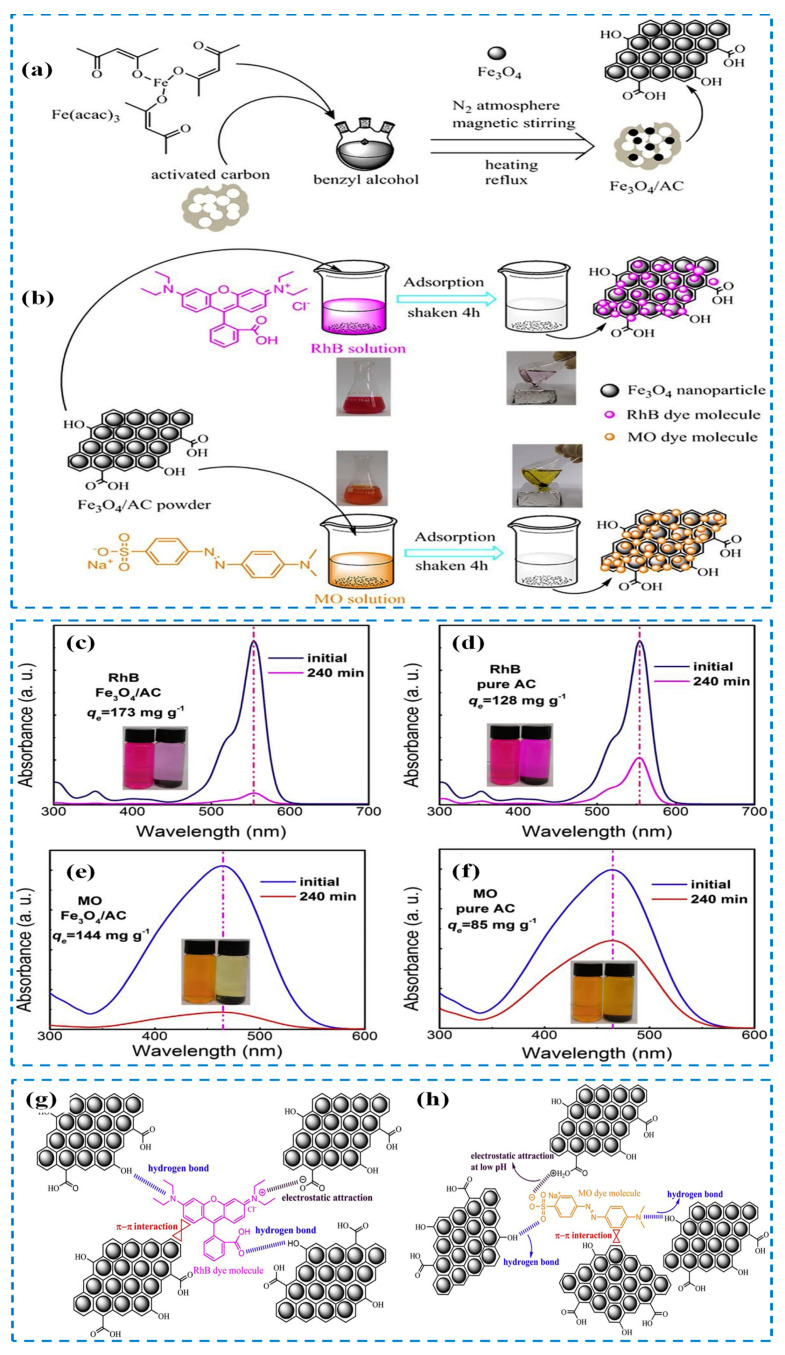
(**a**) Synthesis of the composite magnetic–activated carbon composite, (**b**) removing of RhB and MO from water, (**c**–**f**) showed UV-Vis spectra of RhB and MO adsorption using composite, (**g**) adsorption mechanism of RhB and (**h**) adsorption mechanism of MO [177]. Copyright 2019; reproduced with permission from Elsevier B.V. All rights reserved.

**Figure 12:**
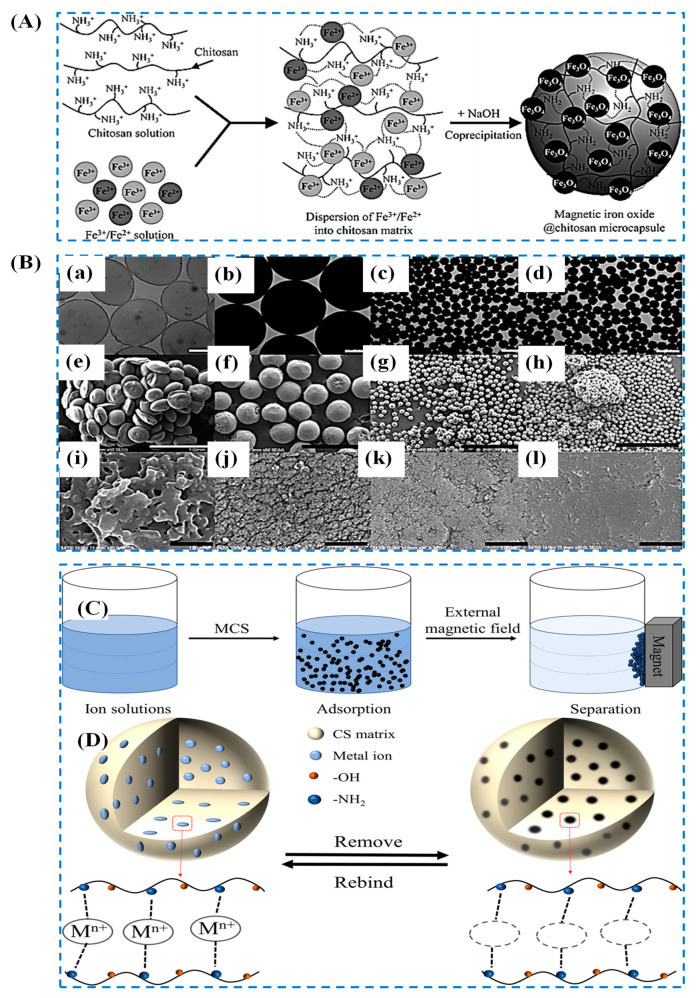
(**A**) Synthetic process of chitosan-based magnetic nanoparticles composite; (**B**) surface morphology change: (a–d) the optical microscope images, (e–l) SEM photographs, (a,e,i) chitosan particles, (b,f,J) magnetic chitosan particles at 3.75 kV, (c,g,k) magnetic chitosan particles at 6 kV, (d,h,l) iron oxide-loaded chitosan particles at 9 kV. Scale bar: (a–d) 200 μm, (e–h) 1 mm, and (i–l) 1 μm. All particles were prepared using 10 wt% NaOH solution [179]. Copyright 2013; reproduced with permission from the Royal Society of Chemistry. (**C**) The magnetic separation process of MCS composites and ion solutions and (**D**) regeneration of ion-imprinted CS composites [180]. Copyright 2021; reproduced with permission from The Author(s), under exclusive license to Springer-Verlag GmbH Germany, part of Springer Nature.

Magnetic adsorbents are gaining popularity in water treatment due to their ability to facilitate adsorption, minimize adsorbent loss, and simplify separation using an external magnetic field. For instance, the magnetic nanocomposite/activated carbon preparation is illustrated in Figure 11a,b. Compared to activated carbon alone, the resulting composite materials exhibit improved adsorption capacity for dyes such as rhodamine B and methyl orange. The maximum dye adsorption capacities were 182.48 mg/g for rhodamine B and 150.35 mg/g for methyl orange, as shown in Figure 11c–f. The adsorption mechanisms are depicted in Figure 11g–h [177]. The synergistic interactions between the composite and Fe_3_O_4_ nanoparticles play a vital role in the dye adsorption strategies of the Fe_3_O_4_/activated carbon composite. The utilization of biopolymer-based nanomagnetic adsorbents is on the rise due to their exceptional adsorption capabilities and high surface area. Among the magnetic materials, Fe_3_O_4_, Fe^0^, γ-Fe_2_O_3_, and MFe_2_O_4_ (M = Cu, Co, Ni and Zn) are well known and commonly employed [178]. Fe_3_O_4_ and γ-Fe_2_O_3_, in particular, are favored due to their strong superparamagnetic properties, ease of production, and low toxicity. Figure 12A illustrates a simple synthetic process for producing chitosan-based Fe_3_O_4_ nanoparticle composites [179].

The magnetism exhibited by these materials plays a crucial role in improving water purification by influencing the physical properties of pollutants. Morphological changes of chitosan-based magnetic nanoparticles are depicted in Figure 12B [179]. Combining these magnetic materials with biopolymers can create composite materials that can be separated magnetically, as shown in Figure 12C [180]. Regeneration tests have been conducted for most magnetic adsorbents, as they can be easily recycled without significant reduction in adsorption capabilities even after 5 to 10 cycles (Figure 12D) [180,181]. Consequently, magnetic-polysaccharide-based adsorbents have garnered significant attention as innovative agents for water purification, offering benefits such as biocompatibility, high performance, low cost, easy separation, and renewability. Combining diverse functionalization and modification techniques enables more efficient removal of mixed pollutants. Organic contaminants are primarily adsorbed through electrostatic interaction, hydrogen bonding, π-π interaction, and ion exchange, while inorganic pollutants are eliminated via electrostatic interaction, complexation, chelation, or ion exchange. The easy separation and reusability of magnetic adsorbents, facilitated by magnetic control, make them highly desirable. Iron oxide nanoparticles, particularly magnetite and maghemite, exhibit superparamagnetic strength and are extensively studied for water purification owing to their large surface area and cost-effectiveness [182].

In contrast, the GO-Fe_3_O_4_-chitosan composites have demonstrated exceptional dye removal capabilities, removing 98% (Q_max_ 249.25 mg/g) of dyes from water [183]. Moreover, the paramagnetic nature of the Fe_3_O_4_ intercalated composite allows for effective adsorption in the presence of a magnetic field [183,184]. Another composite, chitosan/poly (methacrylic acid)/Fe_3_O_4_-GO, has great wastewater purification potential [185]. This composite exhibited a high adsorption capacity with a Q_max_ of 2478 mg/g, efficiently removing MB dye from water within 20 min.

Furthermore, nanocomposites based on guar gum and other biopolymers have emerged as promising materials for removing metal ions and dyes from wastewater [186]. Chitosan and composites have also proven highly effective in absorbing various metal ions and organic dye molecules [180]. Additionally, a magnetic nanoparticle-starch-g-poly (vinyl sulfate) nanocomposite exhibited remarkable adsorption capacities for cationic dyes, with 621 mg/g for MB and 567 mg/g for MG [187]. The sulfate groups on the surface of the adsorbent served as active sites for the adsorption of cationic molecules.

Table 3 provides an overview of recently reported iron-oxide-biopolymer-based polymer nanocomposites investigated for water purification applications. These nanocomposites represent a significant advancement in the field, highlighting their potential for addressing water pollution challenges.

However, even with the successful adsorption of contaminants, there is a concern that some residual pollutants may remain, potentially leading to secondary pollution. To address this issue, developing techniques for completely degrading organic contaminants in water has gained significant attention (catalytic degradation). On the other hand, the membrane filtration approach offers promising possibilities to separate fine particles.

## 5. Membrane Filtration

**Figure 13:**
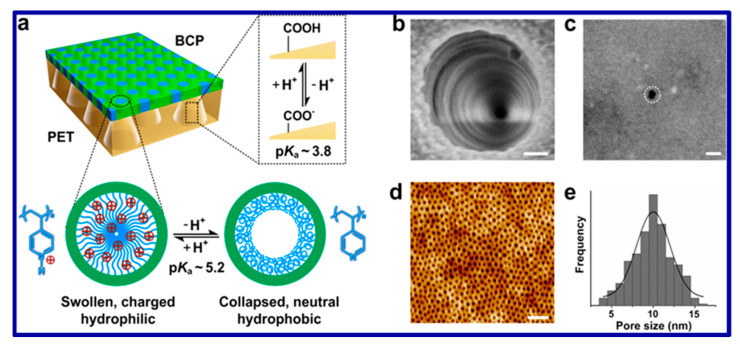
Showcases an engineered asymmetric heterogeneous membrane design. The top layer consists of a pH-responsive porous block copolymer (BCP) membrane (**a**), while the bottom layer is a pH-responsive porous polyethylene terephthalate (PET) membrane with conical nanochannels. SEM images of the base and tip of the conical nanochannels are displayed in (**b**,**c**), respectively, demonstrating a pore density of 107 cm^–2^. The AFM image in (**d**) depicts the BCP membrane atop the PET membrane, with a corresponding histogram showcasing the pore size distribution (pore density: 1011 cm^–2^) and a Gaussian fit (**e**). The scale bars in all images represent 100 nm [215]. Copyright 2015; reproduced with permission from the American Chemical Society.

Membrane filtration techniques are widely used in water treatment to remove contaminants and particles [211]. Two main types of membranes are used in water treatment: symmetric (homogeneous) and asymmetric (heterogeneous) membranes. These membranes differ in terms of pore homogeneity and structure [212,213]. Symmetric membranes comprise a single polymer material, resulting in uniform pore sizes throughout the membrane. On the other hand, asymmetric membranes consist of two distinct layers: a thin outer layer that controls selectivity and a thicker, porous inner layer that provides support [214]. The asymmetric membranes offer higher water flux than symmetric membranes due to their thinner structure, making them suitable for large-scale water purification applications [215]. In addition to symmetric and asymmetric membranes, composite membranes have gained popularity recently. These membranes have a selective layer composed of various inorganic metal oxides. Composite membranes outperform symmetric membranes and even asymmetric membranes in terms of performance. This is mainly attributed to the thin selective layer in composite membranes, which enhances water flux and improves overall membrane efficiency. For example, block copolymers (BCPs) offer great potential for the fabrication of heterogeneous membranes [216]. By manipulating the self-assembled morphologies of BCP membranes, such as their cylindrical and lamellar structures, one- and two-dimensional nanofluidic channels can be readily obtained [217]. The use of macromolecular building blocks in BCPs provides a versatile, engineered, and scalable material system with well-defined pores. In comparison to small molecules, end-tethered polymer brushes offer superior control over ionic transport by increasing the spatial density of functional groups on the channel walls, extending their distribution from two dimensions to three dimensions [217]. A schematic of asymmetric heteraogenious membrane is shown in Figure 13.

**Figure 14:**
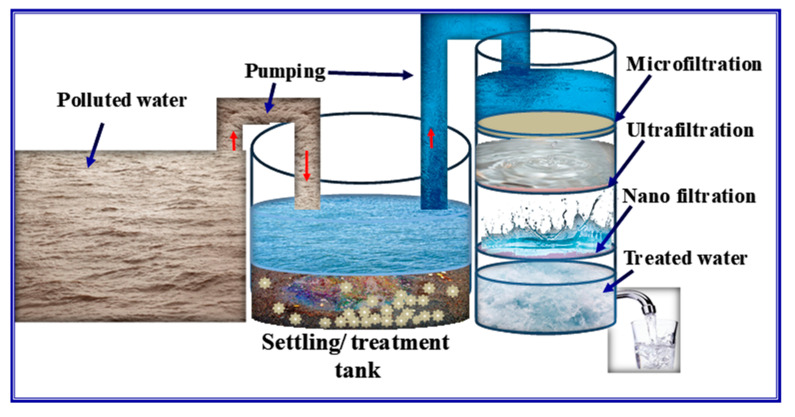
A schematic of membrane filtration process.

On the other hand, membrane filtration techniques, such as microfiltration, nanofiltration, and ultrafiltration, are commonly employed in water treatment processes [218]. The critical distinction among these techniques lies in the pore size of the membranes used [219]. Microfiltration, ultrafiltration, and reverse osmosis remove macroparticles, microparticles, and macromolecules, including inorganic particles, organic colloids, and dissolved natural substances [220]. Nanofiltration, which falls between ultrafiltration and reverses osmosis, operates with a general membrane pore size ranging from 0.5–0.2 nm and 3–8 nm [221]. Additionally, loose nanofiltration (NF) shows great promise for selectively separating dyes and salts, making it a potential candidate for the efficient recovery and cyclic utilization of high-value-added components [222,223]. A schematic of membrane filtration is shown in Figure 14. Membranes for microfiltration and ultrafiltration can be manufactured from both ceramic and polymer materials [224,225]. Ceramic materials offer excellent chemical stability, mechanical strength, ease of cleaning, and long-term durability. However, their brittleness makes large-scale production expensive, which is three times higher than common polymers and challenging. In contrast, polymeric membranes have dominated the market for water purification for a considerable period due to their easy processing and low cost.

One viable solution is the development of ceramic–polymer composite membranes to bridge the significant gap between ceramic and polymeric membranes. These composite membranes are engineered to combine the advantages of both ceramic and polymeric materials, resulting in improved membrane performance, extended lifespan, and additional functionalities [225]. Most membranes used in water treatment, such as cellulose acetate, polyethylene, polytetrafluoroethylene, polypropylene, polyvinylidene fluoride, polyethersulfone, polyacrylonitrile, polysulfone, or other polymers, are made from polymeric materials due to their affordability, good mechanical strength, and operational flexibility [226]. Polyethylene glycol (PEG) and alumina (Al_2_O_3_) is commonly employed to enhance the selectivity and hydrophilicity of membranes as well as to act as a pore-forming agent [227].

However, a significant drawback of membrane filtration is fouling, which refers to accumulating unwanted substances on the surface or within the membrane pores. This leads to reduced filtration efficiency and increased energy consumption [228]. Fouling can occur due to the deposition of particulate matter, scaling, biofilm formation, or adsorption of organic compounds onto the membrane surface [229]. It poses a significant challenge in maintaining the long-term performance and lifespan of the membranes. The membrane’s floor morphology and chemistry influence the fouling of polymer membranes. Membrane sheets of polymers with suitable porosity, chemical and mechanical stability, and high hydrophobicity are typically employed. Natural foulants tend to adsorb onto the membrane surface due to their lyophilic (water attraction) nature [218].

Biopolymer nanocomposites have shown promising results in mitigating fouling in membrane filtration processes [221,229,230,231]. For example, incorporating nanocellulose, chitosan nanoparticles, or graphene oxide nanosheets into biopolymer membranes has improved antifouling properties by reducing the adhesion of foulants and enhancing water permeability [232,233,234]. The recent reports on biopolymer-based graphene oxide nanocomposites aim to explore the synergistic features and diverse applications of these materials, with a particular focus on identifying optimal combinations of biopolymers and carbon nanomaterials for various industrial applications [235,236,237]. The review article thoroughly explores the fabrication, application, and bonding mechanisms of biodegradable biopolymers, such as poly(lactic acid), cellulose, starch, chitosan, alginates, polyamides, and other biodegradable materials, with various forms of graphene, including graphene oxide, reduced graphene oxide, graphene nanoplatelets, etc. [238]. Furthermore, an overview of recent studies is presented, focusing on carbon nanotubes modified with natural polymers (biopolymers) like chitosan, cellulose, and cyclodextrin for water treatment applications. The discussion extends to the cost-effectiveness and economic value of using polymeric hybrid materials based on carbon nanotubes as nano-sorbents for water purification [239]. Notably, these nanocomposite membranes have demonstrated higher flux, improved fouling resistance, and prolonged operational lifetime compared to traditional membranes [240,241,242]. Surface modification techniques, such as grafting or chemical modification, can introduce functional groups that repel foulants or enhance the interaction with specific contaminants, improving separation efficiency and reducing fouling propensity [240]. For example, a PVA–silica nanofiber membrane modified with thiol exhibited superior Cu(II) ion adsorption compared to a PVA nanofiber membrane. The removal of toxic metal ions occurred through electrostatic or chelation mechanisms. PVA/PEI membranes have been utilized for the rejection of Cu(II), Cd(II), Pb(II), and Hg(II) metal ions from wastewater, demonstrating selectivity among different metal ions [243,244]. Similarly, a membrane prepared from PVA and poly (vinyl imidazole) exhibited high rejection ratios, particularly for Hg(II) ions at pH 2.5 [245]. A recent notable study demonstrated a facile surface modification of ultrafiltration (UF) membranes using polydopamine with immobilized nanosilver. The process involved initiating dopamine polymerization on the UF membrane and in situ immobilization of silver and dopamine through a redox reaction, resulting in the formation of a loose separating layer on the membrane’s surface. This loose layer exhibited enhanced fractionation capability compared to a compact layer of polydopamine formed without in situ immobilization of nanosilver and dopamine. The modified membrane showed a remarkable rejection rate of 99.9% for Eriochrome Black T (EBT) and an exceptionally high permeation flux of 39.2 LMH [221]. Table 4 summarizes recent research on membrane filtering employing bio nanocomposites materials.

The development of membrane filtration employing the nanocomposite mentioned above holds great promise for effective water treatment. CNTs/chitosan nanocomposites, lignin–cellulose citrate, polylactic acid-polybutylene succinate-polypropylene carbonate-polyhydroxybutyrate (PLA-PBS-PPC-PHB), poly(urethane)/keratin biofiber, PVA and poly (vinyl imidazole), polyvinyl alcohol/SiO2 composites, nanoclay montmorillonite–chitosan. The utilization of CNTs/chitosan nanocomposites demonstrates a high adsorption capacity for metal ions such as Cu(II), Ni(II), Pb(II), Cd(II), and Co(II), hence contributing to the efficient removal of heavy metal pollutants from water [246]. CNTs’ high surface area provides numerous active sites for metal ion adsorption, while chitosan’s amino groups (–NH_2_) provide binding sites for coordination and chelation. Combining carbon nanotubes and chitosan has a synergistic effect, increasing adsorption efficiency. The nanocomposites’ pH dependency allows for adjustment of adsorption capacity, while their selectivity allows for targeted removal of specific metal ions. Lignin–cellulose-citrate-based membranes have high selectivity for anions and cations, making them suitable for ion separation and purification operations [230]. The functional groups in lignin and cellulose citrate are principally responsible for the membranes’ selectivity. Lignin has phenolic hydroxyl (ߝOH) and carboxyl (ߝCOOH) groups, whereas cellulose citrate has carboxylate (ߝCOO^ࢤ^) groups. Electrostatic interactions, hydrogen bonding, and complexation are all ways these functional groups interact with ions. The carboxylate groups in cellulose citrate can effectively connect with positively charged ions via ion exchange reactions in anions. The phenolic hydroxyl groups in lignin can also contribute to anion binding through hydrogen bonding and complexation. This combination of functional groups improves the membrane’s selectivity for anions, allowing for efficient separation and removal.

The oil–grease and total dissolved solids (TDS) removal characteristics of the multi-component polymer mix PLA-PBS-PPC-PHB make it appropriate for treating oily wastewater [150]. Poly(urethane)/keratin biofiber membranes remove Cr(VI) ions well, indicating their potential for treating chromium-contaminated water [247]. PVA and poly (vinyl imidazole) membranes are effective for mercury removal from water sources due to their high Hg(II) removal effectiveness [245]. The high performance of these membranes in mercury removal can be attributed to the affinity of imidazole groups towards metal ions, including Hg(II). Imidazole groups possess lone pair electrons on the nitrogen atom, which can form coordination bonds with metal ions through complexation. These interactions allow the membrane to capture and retain mercury ions effectively. Furthermore, the hydroxyl groups in PVA can also contribute to removing mercury ions through hydrogen bonding and complexation. The hydroxyl groups can form strong interactions with Hg(II), promoting its adsorption and retention on the membrane surface. Polyvinyl alcohol/SiO_2_ composites exhibit good Cu(II) adsorption capacity, contributing to improved water purification [248]. It is due to the synergistic effect between PVA and SiO_2_, resulting from their respective chemical properties. Combining the hydroxyl groups in PVA and the adsorption sites provided by SiO_2_ nanoparticles creates a synergistic effect, enhancing the overall adsorption capacity for Cu(II) ions. The hydroxyl groups of PVA and the silanol groups of SiO_2_ work cooperatively to capture and bind Cu(II) ions, increasing the efficiency of water purification processes. Integrating nanoclay montmorillonite-chitosan in membranes results in excellent COD removal, demonstrating its potential for treating organic contaminants in wastewater [249]. Chitosan-linked activated carbon–nano-bentonite membranes have a high polyaromatic hydrocarbon (PAH) removal efficiency, making them appropriate for removing organic pollutants. TiO_2_-loaded fly ash chitosan composite membranes show good dye removal efficacy for Congo red and Methylene blue, suggesting a potential solution for dye-contaminated water [251]. The adsorption process of chitosan successfully catches and maintains the dye molecules on the membrane surface via electrostatic interactions and hydrogen bonding. The synergistic action of TiO_2_ and chitosan results in increased water filtration performance for dye-contaminated water.

## 6. Limitation, Challenges, and Opportunities

Biopolymer-based nanocomposites, incorporating adsorbents like graphene oxide, carbon nanotubes, and nanoclays, offer significant potential for water treatment applications [232,233,234]. However, certain challenges need to be addressed to fully realize their benefits. These include scalability and cost-effectiveness in large-scale production, improved nanocomposite stability and reusability for long-term performance, and efficient nanofiller dispersion within the biopolymer matrix [252]. Environmental sustainability requires careful consideration of nanocomposite regeneration and used adsorbent disposal. Despite these obstacles, opportunities exist for innovation through research and development, leading to advanced nanocomposite materials and enhanced synthesis methods [238].

Combining magnetic adsorbents [253] and membrane filtration technology can enhance adsorption and separation efficiency, leading to more effective water treatment solutions [254]. Furthermore, integrating multifunctional and responsive biopolymer-based nanocomposites with current water treatment technologies opens up possibilities for energy-efficient and on-demand water purification systems [252].

Collaboration among researchers and industry stakeholders is crucial for advancing these solutions, ensuring long-term and affordable water treatment. Research on complementary methods and real industrial effluent testing will further enhance water purification effectiveness.

## 7. Conclusions

Biopolymer-based nanocomposites with adsorbents such as graphene oxide, carbon nanotubes, and nanoclays have much potential for water treatment applications. These nanocomposites offer better adsorption capacities, improved membrane performance, and efficient pollutant removal from water sources. Using nanomaterials and magnetic adsorbents in conjunction with membrane filtration methods yields promising results in improving efficiency and selectivity in water purification operations. However, there are obstacles to solve, such as scalability, cost-effectiveness, stability, and regeneration of nanocomposites and disposal of used adsorbents. Nonetheless, these issues can be overcome with additional research and innovation, creating sustainable and effective water treatment systems. Integrating biopolymer-based nanocomposites with sophisticated water treatment technologies like responsive materials and hybrid systems offers new possibilities for on-demand and energy-efficient water purification systems. Researchers can promote the growth of biopolymer-based nanocomposites and contribute to the development of complete and sustainable water treatment techniques by collaborating across disciplines and involving industry partners.

## Figures and Tables

**Table 3:** Iron-oxide-biopolymer-based polymer nanocomposites are used for water purification.

Sl. No	Adsorbent/Flocculent	Pollutant	Results (% or Q_max_ mg/g)	Ref.
1.	Fe_3_O_4_-Starch-g-PVA	MB MG	621 mg/g 567 mg/g	[187]
2.	Starch-coated Fe_3_O_4_ Nanoparticles	Textile dye Optilan Blue	74.05 mg/g	[188]
3.	GO–Fe_3_O_4_ hybrid composite	MB Natural red	167.2 mg/g 171.3 mg/g	[189]
4.	Chitosan/Fe–hydroxyapatite beads	MB	1324 mg/g	[190]
5.	Fe_3_O_4_/β-cyclodextrin/GO	MG	740.7 mg/g	[191]
6.	Magnetic chitosan nano-adsorbent	Cu Cd Zn	99.9% 95.0% 81.7%	[192]
7.	Magnetic mesoporous silica–chitosan composite	Hg (II)	437.8 mg/g	[193]
8.	Carboxymethyl-chitosan-based magnetic	Ciprofloxacin	527.9 mg/g	[194]
9.	Anionic-polyacrylamide-modified chitosan magnetic composite	MB	1044.1	[195]
10.	Manganese ferrite nanoparticles covered with carboxymethyl starch	Pb (II)	34–64 mg/g	[196]
11.	Sodium alginate/CMC/Fe_3_O_4_	Mn (II), Pb (II), Ni (II)	71.83, 89.49, 105.93 mg/g	[197]
12.	β-cyclodextrin-Fe_3_O_4_/MWCNT	Ni (II)	103 mg/g	[198]
13.	Fe_3_O_4_/wood biochar	Acid orange and Cr (VI)	110.27, 80.96 mg/g	[199]
14.	GO/Fe_3_O_4_/glucose	U (VI)	390.70 mg/g	[200]
15.	Cellulose/polyethyleneimine-Fe_3_O_4_	Hg (II)	247.51 mg/g	[201]
16.	Chitosan activated carbon-Fe_3_O_4_ composites	Cr (VI)	99.8%	[174]
17.	Magnetic chitosan−GO nanocomposites	MB	95.31 mg/g, pH 5.3, 303K	[202]
18.	GO-Fe_3_O_4_-chitosan composites	MB	98.0%	[183]
19.	Chitosan/poly (methacrylic acid)/Fe_3_O_4_ -GO composite	MB	2478 mg/g	[185]
20.	Lignin-grafted magnetic nanocomposite	Fluoroquinolone	108.2 mg/g	[203]
21.	Lignin magnetic composites	Malachite green	456.62 mg/g	[204]
22.	Cellulose nanofiber–GO magnetic composite	Methylene blue	83.53%	[205]
23.	Magnetic-cellulose-based ionic liquid	Congo red and methyl blue	1299.3 and 1068.1 mg/g	[206]
24.	Fe_3_O_4_–chitosan composite	Methyl orange	638.6 mg/g	[207]
25.	Dragon fruit biopolymer–CoFe_2_O_4_	Ni (II)	88%	[208]
26.	Inulin–Fe_3_O_4_ nanocomposite	Co (II), Cu (II), Hg (II)	152.5, 167.7, 198.0 mg/g	[209]
27.	Fe3O4–chitosan–PAMm composite	Food dye	359.71 mg/g	[210]

**Table 4:** Recent reports of water purification using membrane filtration.

Sl No	Membrane	Pollutants	Optimum Rejection (%)	Pressure (bar)	pH	Ref.
1.	CNTs/chitosan biopolymer nanocomposite	Cu(II), Ni(II), Pb (II), Cd(II), and Co(II) ions.	71–92.2	22	3.0	[246]
2.	Lignin–cellulose citrate	Anions Cations	12–42 27–54	-	>7.0	[230]
3.	Polylactic acid-polybutylene succinate-polypropylene carbonate-polyhydroxybutyrate.	Oil–grease TDS	98.6 89.15	1 4	-	[150]
4.	Poly (urethane)/keratin biofiber	Cr (VI)	38.0 11.0	0.7	>8–<4	[247]
5.	PVA and poly (vinylimidazole)	Hg (II)	99.4	3.0	2.5	[245]
6.	Polyvinyl alcohol/SiO_2_ composites	Cu (II)	93.1	-	5–6	[248]
7.	Nanoclay montmorillonite-Chitosan	COD	65.7	-	-	[249]
8.	Chitosan-linked activated carbon–nano-bentonite	Polyaromatic hydrocarbons	99.3	-	6.0	[250]
9.	TiO_2_ loaded fly ash chitosan composite	Congo red Methylene blue	98.0 55.7	-	1.8	[251]
10.	Polydopamine-functionalized polysulfone membrane	Eriochrome Black T	99.9	1–3	-	[221]

## Data Availability

Not applicable.
